# Intersections and Challenges in the Management of Acute Coronary Syndrome and Stroke: Pathophysiology, Treatment Dilemmas, and Integrated Prevention Strategies

**DOI:** 10.3390/jcm14072354

**Published:** 2025-03-29

**Authors:** Maria Cristina Carella, Eugenio Carulli, Francesco Loizzi, Simona Quarta, Alessandra Freda, Paolo Basile, Fabio Amati, Marco Maria Dicorato, Michele Davide Latorre, Maria Ludovica Naccarati, Cosimo Daniele Lenoci, Sebastiano Cicco, Gianluca Pontone, Cinzia Forleo, Andrea Igoren Guaricci, Marco Matteo Ciccone, Vincenzo Ezio Santobuono

**Affiliations:** 1 Cardiology Unit, Interdisciplinary Department of Medicine (DIM), University of Bari “Aldo Moro”, University Hospital Consortium Polyclinic of Bari, Piazza G. Cesare 11, 70124 Bari, Italypaolo.basile@uniba.it (P.B.); m.dicorato20@studenti.uniba.it (M.M.D.); daniele.lenoci01@gmail.com (C.D.L.); cinzia.forleo@uniba.it (C.F.); marcomatteo.ciccone@uniba.it (M.M.C.); vincenzoezio.santobuono@uniba.it (V.E.S.); 2 Cardiology Unit, SS Annunziata Hospital, 74100 Taranto, Italy; 3 Neurology Unit, Ospedale della Murgia “Fabio Perinei”, 70022 Altamura, Italy; 4 Unit of Internal Medicine “Guido Baccelli” and Unit of Hypertension “A.M. Pirrelli”, Department of Precision and Regenerative Medicine and Ionian Area—(DiMePRe-J), University of Bari “Aldo Moro”, University Hospital Consortium Polyclinic of Bari, Piazza G. Cesare 11, 70124 Bari, Italy; 5 Centro Cardiologico Monzino IRCCS, 20138 Milan, Italy; 6 Department of Biomedical, Surgical and Dental Sciences, University of Milan, 20138 Milan, Italy

**Keywords:** acute coronary syndrome, stroke, intracerebral hemorrhage, myocardial infarction, dual antiplatelet therapy, anticoagulation therapy, percutaneous coronary intervention

## Abstract

Acute coronary syndrome (ACS) and stroke are interconnected conditions that often share risk factors such as atherosclerosis, thrombosis, and systemic inflammation. When these events occur simultaneously, they present unique diagnostic and therapeutic challenges. This review explores the pathophysiological mechanisms linking ACS and stroke, including common pathways like plaque instability, cardioembolism, and endothelial dysfunction, while highlighting the distinct features of ischemic and hemorrhagic strokes. The manuscript provides an overview of diagnostic strategies, emphasizing the role of biomarkers, advanced neuroimaging, and risk stratification tools in guiding acute management. Furthermore, the review delves into treatment approach, emphasizing the need to balance reperfusion therapies for ACS with thrombolysis or thrombectomy for ischemic stroke while carefully managing the challenges posed by anticoagulation in cases complicated by bleeding. Long-term strategies for secondary prevention are examined, including antithrombotic regimens tailored to the dual risk of thrombosis and bleeding, as well as lipid-lowering and blood pressure management. Future perspectives highlight the potential of novel pharmacological agents, neuroprotective therapies, and AI-driven tools to enhance patient outcomes. This review underscores the importance of integrated, multidisciplinary care and identifies key areas for future research to optimize the management of these high-risk patients.

## 1. Introduction

Cardiovascular diseases constitute the leading cause of death in the Western world, accounting for approximately 18.6 million deaths globally in 2019 (95% uncertainty interval (UI): 17.1–19.7 million). The prevalence of cardiovascular diseases has nearly doubled over the past three decades, increasing from 271 million (95% UI: 257–285 million) in 1990 to 523 million (95% UI: 497–550 million) in 2019 [1,2]. The main pathological condition responsible for the high mortality is atherosclerosis. Comorbidities such as diabetes and obesity substantially increase the burden of cardiovascular disease, both by exacerbating the progression of atherosclerosis and by amplifying the risk of adverse cardiovascular outcomes. The Global Burden of Disease 2019 study reported that high body mass index was responsible for 5.02 million (95% UI: 3.57–6.54 million) disability-adjusted life years (DALYs) due to ischemic heart disease globally in 2019, while diabetes mellitus accounted for 2.04 million (95% UI: 1.56–2.53 million) DALYs [2]. These findings underscore the critical need for targeted interventions addressing metabolic risk factors to reduce cardiovascular morbidity and mortality. Clinical manifestations are distinguished into chronic and acute and primarily affect by high incidence the coronary and cerebral district [3]. Ischemic heart disease and stroke remain the most significant contributors to cardiovascular mortality, with ischemic heart disease alone responsible for 9.14 million deaths (95% UI: 8.40–9.74 million) and stroke for 6.55 million deaths (95% UI: 6.00–7.02 million) in 2019 [2]. In order to contain the social and economic impact of acute clinical manifestations and the progression of chronic forms, it is essential to optimize the diagnostic–therapeutic pathways of acute events distinctly from the slowly developing and progressive forms [4,5,6,7,8].

Stroke is a known acute coronary syndrome (ACS) complication and remains closely associated with elevated mortality rates [9,10,11]. This association is particularly pronounced when stroke occurs within the first year after myocardial infarction (MI), with intracranial hemorrhage (ICH) often linked to an even higher risk of mortality [10,12,13]. The incidence of post MI stroke has been estimated at 0.5–2.1% during hospitalization and 0.7–2.1% within the first 30 days post-MI [14,15]. This elevated risk tends to persist through the first year, with an incidence rate of approximately 1.1–4.1% [16]. Recent decades have witnessed a reduction in both the incidence and mortality rates of post-MI stroke, largely attributable to timely interventions and improvements in medical care [17,18,19].

In particular, the novelty in anticoagulation and antithrombotic strategies in secondary prevention may influence the proportion of ICH cases within the total stroke incidence, with ischemic stroke (IS) still representing the majority of post-MI stroke events [13,20,21]. This shift underscores the importance of optimizing medical practices to balance effective secondary prevention with the minimization of bleeding risks.

**Figure 1:**
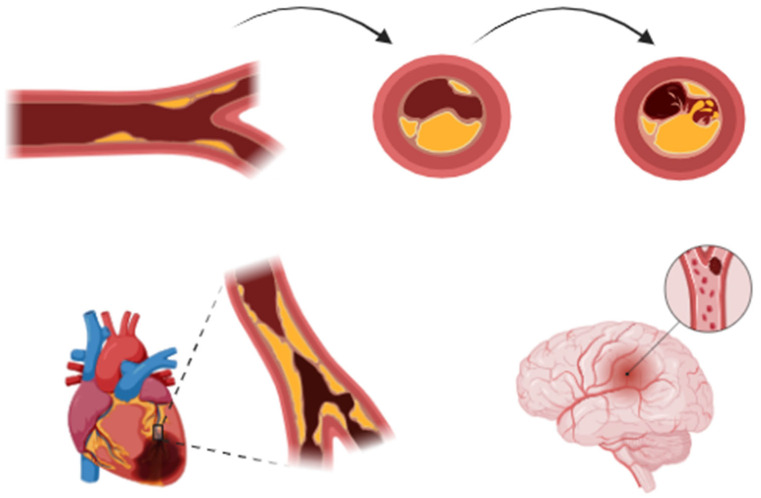
Progression of atherosclerosis: from formation of a stable fibrous cap to development of a vulnerable plaque prone to rupture, leading to thrombotic complications. This shared pathophysiological mechanism underlies both acute coronary syndromes and ischemic stroke, particularly large-artery atherosclerosis.

This narrative review explores the intersection of ACS and stroke, with a focus on shared pathophysiological mechanisms and prevention strategies. The primary objective is to analyze whether current management strategies are adequate to reduce the risk of cerebrovascular events in patients with ACS and vice versa. The subsequent discussion will address the question of integrated strategies that have the potential to enhance the prevention and concurrent management of ACS and stroke (Figure 1).

We will examine both shared and unique risk factors and address the current approaches to treatment and management. By analyzing recent data and evolving practices, we aim to clarify the implications for both acute and long-term care, ultimately enhancing our understanding of how best to mitigate the risks associated with these critical cardiovascular events.

## 2. Pathophysiology of Acute Coronary Events and Stroke

### 2.1. Definition and Classification of Acute Myocardial Infarction

ACS encompasses a range of conditions marked by recent alterations in symptoms or clinical signs, which may be accompanied by electrocardiogram (ECG) changes and/or elevations in cardiac troponin (cTn) levels [9,22]. Within this spectrum, patients with suspected ACS can ultimately be diagnosed with either acute myocardial infarction (AMI) or unstable angina (UA) [9]. According to the Fourth Universal Definition of Myocardial Infarction, AMI involves acute myocardial injury in the presence of ischemia, confirmed by a measurable rise or fall in cTn values, with at least one reading above the 99th percentile upper reference limit (URL), in conjunction with additional evidence of ischemia [23]. The criteria for AMI diagnosis include ischemic symptoms, such as chest pain or discomfort, new ischemic changes observed on the ECG, development of pathological Q waves, imaging that shows new loss of viable myocardium or new regional wall motion abnormality consistent with an ischemic origin; detection of coronary thrombosis through angiography or autopsy.

**Figure 2:**
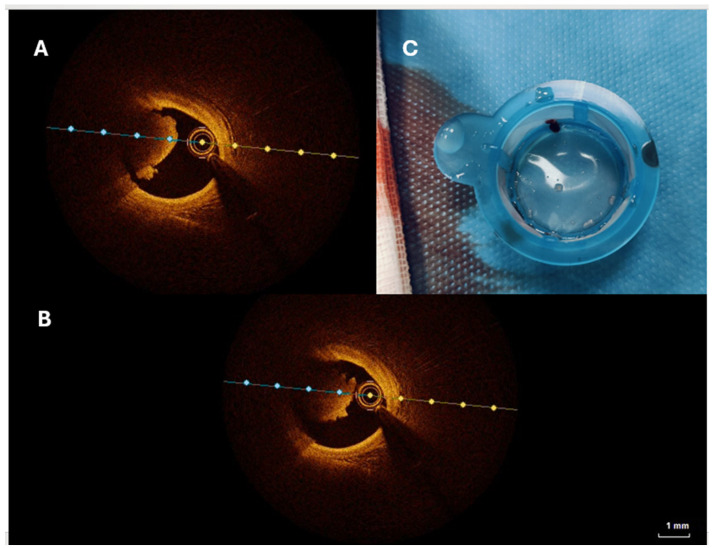
Intravascular and procedural findings in a STEMI patient with left anterior descending artery (LAD) involvement. (**A**,**B**) Optical coherence tomography (OCT) images revealing the presence of intracoronary thrombus in the LAD. (**C**) Aspirated coronary thrombus retrieved from a STEMI patient with LAD occlusion.

UA, by contrast, indicates myocardial ischemia at rest or minimal exertion, without markers of acute cardiomyocyte injury or necrosis [23]. Clinically, UA is identified by prolonged chest pain at rest (>20 min), new onset of severe angina, or angina that increases in frequency, duration, or occurs following a recent MI episode [9]. The clinical presentations of ACS therefore range from transient ischemic episodes in UA to definitive myocardial infarction in AMI. AMI is classified into two main types:

- STEMI (ST-Segment Elevation Myocardial Infarction): Defined by persistent ST-segment elevation on the ECG, typically indicating complete blockage of a coronary artery (Figure 2).

- NSTEMI (Non-ST Segment Elevation Myocardial Infarction): Occurs without persistent ST-segment elevation and is often associated with a partial blockage of a coronary artery.

### 2.2. Incidence and Risk of Stroke Following Acute Coronary Syndrome

The risk of stroke in AMI patients is influenced by factors such as AMI type, patient comorbidities, and treatment interventions. Studies indicate a stroke incidence rate of approximately 0.7% to 2.2% in patients hospitalized for STEMI or NSTEMI [10,24,25] (Table 1). Stroke following AMI is predominantly ischemic, accounting for 90% of cases [10,14,19,20].

Long-term data reveal varying stroke incidence post-AMI:

- From the time of AMI hospitalization to one-month post-discharge, stroke incidence is estimated at 1.1–1.5% [15].
- At six months, the incidence is approximately 3% [11,26].
- At ten years, this rate increases to around 8.6%, though these data are primarily derived from smaller studies and specific registries [16,27].

A recent large-scale analysis of the National Inpatient Sample (NIS) database in the USA from 2000 to 2017 examined 11,622,528 AMI admissions, revealing that 1.6% of these cases had concomitant acute ischemic stroke [26]. A breakdown of these data highlights:

- A slight increase in ischemic stroke rates among STEMI patients (adjusted odds ratio, 1.10 [95% CI, 1.04–1.15]) over the study period.
- A notable decrease in ischemic stroke rates in NSTEMI patients (adjusted odds ratio, 0.47 [95% CI, 0.46–0.49]) (*p* < 0.001).

Patients with concomitant stroke were generally older, more often female, of non-white race, and exhibited higher rates of comorbidities and arrhythmias. Furthermore, this cohort underwent coronary angiography (46.9% vs. 63.8%) and percutaneous coronary intervention (PCI) (22.7% vs. 41.8%) less frequently compared to AMI patients without stroke, underscoring potential disparities in access to invasive management (*p* < 0.001).

**Table 1:** Key studies analyzing incidence of stroke in context of acute coronary syndromes.

Study	Population	Stroke Incidence	Key Findings
Hurskainen et al. (2022) [10]	Patients with ACS	0.7–2% during hospitalization	90% of strokes post-AMI are ischemic
Saczynski et al. (2008) [11]	Patients post-AMI	1.1–1.5% at one month	Incidence of stroke peaks in the first 30 days post-MI
Ulvenstam et al. (2014) [16]	Post-AMI patients	3% at six months	Stroke risk persists beyond hospitalization
Sundboll et al. (2016) [27]	AMI survivors	8.6% at ten years	Long-term stroke risk remains substantial
Aggarwal et al. (2021) [26]	National Inpatient Sample (USA) 2000–2017	1.6% during hospitalization	STEMI patients had higher ischemic stroke rates
Hachet et al. (2014) [14]	Post-MI patients	0.5–2.1% during hospitalization	In-hospital stroke incidence varies with MI severity and treatment strategies
Westerhout et al. (2006) [15]	NSTEMI patients	0.7–2.1% within 30 days	Stroke risk in NSTEMI patients is influenced by treatment approaches and patient comorbidities

### 2.3. Pathophysiological Mechanisms of Stroke in Acute Coronary Syndrome Patients

The pathophysiology of both ACS and stroke involves complex atherosclerotic and thrombotic processes, although significant distinctions exist between the two conditions. In acute coronary events, particularly MI, the primary mechanism is plaque rupture within coronary arteries, leading to the formation of a thrombus that limits blood flow, resulting in ischemia and subsequent myocardial damage [28,29]. Contributory factors include inflammation, endothelial dysfunction, and hypercoagulability, which collectively heighten the risk of plaque instability and rupture [9,30,31]. The role of oxidative stress and endothelial dysfunction in linking acute coronary syndromes and stroke requires greater attention. Studies have demonstrated that oxidative stress plays a crucial role in plaque instability and cerebrovascular damage. Altered nitric oxide production, excessive generation of reactive oxygen species, and the activation of pro-inflammatory pathways are key factors contributing to vascular injury progression at both coronary and cerebral levels [31]. Furthermore, impaired endothelial function is a major determinant of increased thrombotic risk and the progression of atherosclerotic lesions, thereby facilitating acute events such as myocardial infarction and stroke [31].

**Figure 3:**
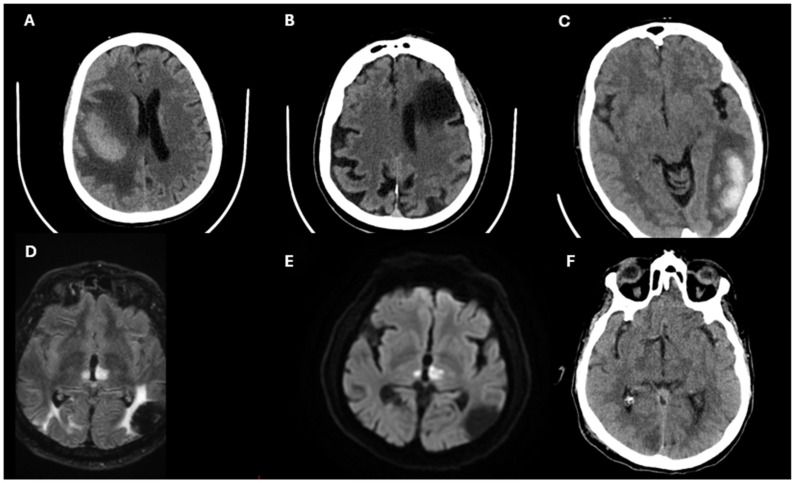
Representative neuroimaging findings of different types of strokes. (**A**) Right frontoparietal intraparenchymal hemorrhage with surrounding perilesional edema, ventricular compression, and midline shift (CT). (**B**) Sequela of ischemic stroke in left frontal region (CT). (**C**) Left temporo-occipital intraparenchymal hemorrhage with perilesional edema (CT). (**D,E**) Subacute bilateral thalamic ischemic stroke shown on FLAIR (Fluid-Attenuated Inversion Recovery) (**D**, left) and DWI (Diffusion-Weighted Imaging) ((**E**), right) MRI sequences. (**F**) Ischemic stroke in right occipital region (CT).

Similarly, IS is often due to atherosclerotic disease or cardioembolism [32] (Figure 3).

However, while MI is usually attributed to localized coronary plaque rupture, IS’s embolic sources are material freed from heart due to atrial fibrillation (AF) or left ventricular dysfunction, which increase itself thrombus formation in the cardiac chambers [33,34]. Recent studies show a bidirectional relationship where post-MI changes, such as wall motion abnormalities, can predispose patients to embolic stroke, linking the two conditions further [33,35].

ICH represents a distinct pathophysiological entity, primarily driven by hypertension-induced vascular damage, resulting in vessel rupture [36]. While atherosclerosis is not a direct contributor to ICH, anticoagulant and antithrombotic therapies used post-MI to prevent ischemic complications may inadvertently elevate ICH risk, complicating therapeutic strategies [9,37].

Emerging evidence suggests that oxidative stress, endothelial dysfunction, and systemic inflammation are shared pathological factors that exacerbate both ACS and stroke risk, though they may manifest through different vascular mechanisms [38,39,40,41]. This overlap highlights the need for integrated approaches targeting both cardiovascular and cerebrovascular protection. Understanding these mechanisms not only improves the characterization of MI and stroke but also aids in identifying patients at high risk of both events, guiding secondary prevention strategies.

### 2.4. Atrial Fibrillation in the Setting of Acute Coronary Syndromes

AF is the most frequent supraventricular arrhythmia in patients with ACS, with an incidence ranging from 2% to 23% [42]. The risk of new-onset AF is significantly increased in patients with MI, with estimates suggesting a 60–77% higher risk compared to those without MI [43]. The coexistence of AF and ACS is associated with an increased likelihood of STEMI and non-STEMI ACS, as well as a greater burden of comorbidities and a higher risk of in-hospital complications and adverse long-term outcomes [44,45].

AF in the setting of ACS may arise due to several mechanisms, including myocardial ischemia, autonomic imbalance, systemic inflammation, and increased left atrial pressure due to ventricular dysfunction. In some cases, transient and self-terminating AF episodes during ACS may serve as a predictor of long-term stroke risk [45]. Moreover, AF is a known precipitant of type 2 MI, further complicating the clinical picture and therapeutic decision-making [46].

The presence of AF in ACS necessitates careful management, particularly in the context of antithrombotic therapy. The need for both oral anticoagulation to prevent thromboembolism and dual antiplatelet therapy (DAPT) to reduce ischemic events lead to an increased risk of bleeding [47,48]. Current guidelines recommend a short-term triple therapy regimen, consisting of oral anticoagulation, aspirin, and a P2Y12 inhibitor, in patients undergoing PCI. The duration of triple therapy should be minimized, with a general recommendation to limit it to ≤1 week in most patients to reduce bleeding risk [45]. For high-risk ischemic patients, such as those with STEMI, prior stent thrombosis, complex coronary interventions, or prolonged cardiac instability, extending triple therapy up to one month may be considered [49]. Beyond the initial period, dual therapy with oral anticoagulation and a P2Y12 inhibitor is preferred over prolonged triple therapy due to its lower bleeding risk, with clopidogrel being the recommended P2Y12 inhibitor given its favorable safety profile compared to ticagrelor and prasugrel [50,51].

In patients with ACS who are not undergoing revascularization, a single antiplatelet agent combined with a long-term direct oral anticoagulant (DOAC) is generally sufficient to minimize bleeding risk. For those with stable coronary artery disease beyond 12 months, monotherapy with oral anticoagulation alone is adequate, and additional antiplatelet therapy is not required [52]. Proton pump inhibitors should be considered for gastrointestinal bleeding prevention in patients receiving combined antithrombotic therapy.

AF in ACS is associated with worse short- and long-term outcomes, including higher mortality, increased rates of heart failure, and an elevated risk of thromboembolic events [45]. Therefore, an individualized approach balancing ischemic and bleeding risks is essential to optimize outcomes in this complex patient population.

### 2.5. Cardioembolic Mechanisms and Procedural Risk in Ischemic Stroke Following Myocardial Infarction

The cardiac mechanisms contributing to ischemic stroke in patients with recent MI are multifaceted, encompassing abnormalities in the heart wall and chambers, valve disorders, and arrhythmias [53]. Among these, left ventricular thrombus (LVT), reduced left ventricular ejection fraction (LVEF), and wall motion abnormalities (WMA) stand out as significant contributors [54,55]. These factors highlight the complex interplay between structural and functional cardiac changes and embolic risk.

Cardioembolism remains a primary cause of ischemic stroke in patients with recent MI. According to the TOAST Trial classification, conditions such as acute MI (<4 weeks), LVT, and akinetic ventricular segment disease are high-risk sources of cardioembolism [54]. In contrast, hypokinetic left ventricular segments or MI occurring beyond 4 weeks but within 6 months are categorized as medium-risk sources [54]. Aggressive reperfusion strategies in MI have reduced the incidence of ischemic stroke by saving myocardium and mitigating WMA, thereby lowering embolic potential [26].

LVT formation is most common within the first two weeks following anterior STEMI, with incidence rates varying between 4% and 39% [55,56]. Predictors of LVT include extensive infarct size, apex involvement, severe diastolic dysfunction, and reduced LVEF [56]. Advanced imaging techniques, such as contrast-enhanced echocardiography and cardiac magnetic resonance, enhance the detection of LVT and optimize risk stratification [57,58].

MI-induced WMAs are a critical factor for embolic stroke, as they contribute to blood stasis and thrombus formation [59]. Reduced LVEF, independently, is associated with an increased stroke risk, with the likelihood of ischemic events inversely proportional to LVEF [60]. 

AF, frequently complicating acute MI, is a key predictor of ischemic stroke [17,33,45]. Both new-onset and transient AF during STEMI have been linked to an elevated risk of embolic events during follow-up [17,33,45]. Oral anticoagulation is generally recommended in AF patients regardless of echocardiographic findings, emphasizing its importance in stroke prevention.

### 2.6. Procedural Risks in Stroke Development

Interventional procedures for MI, such as PCI and coronary artery bypass grafting (CABG), carry an intrinsic risk of ischemic stroke due to embolic mechanisms. The incidence of stroke ranges from 0.07% to 0.4% with PCI and 1.5% to 3% with CABG [61,62,63,64,65].

Embolic strokes following PCI can arise from the dislodgement of calcified plaque debris, thrombus fragments, or material from atherosclerotic lesions [61,62,64]. Thrombus aspiration, although intended to mitigate risk, has occasionally been associated with embolization when incomplete aspiration releases residual thrombus in the systemic circulation [61,62,64]. The transradial approach slightly increases the risk of ischemic stroke compared to transfemoral due to the anatomical proximity of the subclavian artery to the carotid and vertebral arteries [61,62,64].

Stroke following CABG often occurs perioperatively, driven by embolism from aortic manipulations or hemodynamic instability [63,65]. Perioperative AF, arterial dissections, and hypotension further contribute to stroke risk [63,65]. Despite these risks, early revascularization through PCI or CABG has demonstrated an overall reduction in long-term stroke incidence, likely due to improved myocardial recovery and reduced thrombotic burden [63,65].

## 3. Diagnosis and Biomarkers in Acute Coronary Syndrome and Stroke

Accurate and timely diagnosis is crucial in both ACS and stroke to improve outcomes and reduce mortality. For ACS, the combination of clinical evaluation, biomarkers and advanced imaging plays a pivotal role in confirming the diagnosis, while in stroke, neuroimaging and additional diagnostic tools are essential for differentiating ischemic from hemorrhagic subtypes.

### 3.1. Diagnostic Approach in Acute Coronary Syndrome

In ACS, cardiac biomarkers, particularly cTn, are the cornerstone of diagnosis. Elevated cTn levels, specifically above the 99th percentile URL, indicate myocardial injury, with a rise and/or fall pattern further confirming acute ischemic events. The high sensitivity and specificity of high-sensitivity troponin assays (hs-cTn) have transformed the diagnostic process, enabling early identification of even minor myocardial damage [23,66].

ECG remains the first-line diagnostic tool, distinguishing between STEMI and NSTEMI/UA. New persistent ST-segment elevation confirms STEMI, while ischemic changes without ST-segment elevation, such as T-wave inversions or ST depressions, support NSTEMI or UA diagnosis [9,37]. Advanced imaging, including echocardiography and cardiac MRI, provides valuable information on myocardial viability, regional wall motion abnormalities, and infarct size, which can guide management decisions [67,68,69,70,71,72,73,74].

Coronary angiography remains the gold standard for assessing coronary artery anatomy and guiding revascularization procedures. However, its primary limitation lies in its two-dimensional nature, which precludes an accurate characterization of atherosclerotic lesions and the pathophysiological mechanisms underlying ACS. Intravascular imaging has revolutionized ACS management by enabling a more detailed evaluation of plaque characteristics and vulnerability. The two most used modalities are intravascular ultrasound (IVUS), which provides insights into plaque burden and vascular morphology, with an axial resolution of 100–200 µm, allowing for the identification of fibrocalcific plaques and accurate vessel sizing, and, on the other hand, optical coherence tomography (OCT), which offers superior spatial resolution (~10 µm), enabling detailed morphological assessment of plaques and distinguishing between plaque rupture, plaque erosion, and calcified nodules [75]. In particular, OCT plays a crucial role in identifying the different types of plaques that contribute to the pathogenesis of ACS [76]. Among these, vulnerable plaques, also known as thin-cap fibroatheromas, are characterized by a large lipid core covered by a thin fibrous cap (<65 µm), often infiltrated by macrophages [77]. These plaques are highly prone to rupture and are considered a major precursor of thrombotic events.

Another key mechanism underlying ACS is plaque rupture, which is most commonly associated with ST-segment elevation myocardial infarction (STEMI). This process involves the disruption of the fibrous cap, leading to the exposure of the lipid-rich necrotic core to the bloodstream, which in turn triggers thrombus formation [76,77]. A distinct yet equally important mechanism is plaque erosion, which accounts for approximately 30% of ACS cases [78]. Unlike plaque rupture, plaque erosion is characterized by endothelial loss without overt disruption of the fibrous cap. This mechanism is often observed in younger patients and women, and it tends to result in thrombus formation without extensive lipid-rich plaque involvement.

Finally, eruptive calcified nodules, although less frequent, represent another substrate for ACS, particularly in elderly patients with advanced coronary artery disease [78]. These lesions are formed by calcified deposits protruding into the arterial lumen, which can lead to thrombosis due to their irregular surface and mechanical stress on the vessel wall. Through its superior resolution, OCT enables precise differentiation of these distinct plaque phenotypes, thereby improving diagnostic accuracy and guiding targeted therapeutic strategies in ACS management.

### 3.2. Diagnostic Approach in Stroke

Stroke diagnosis hinges on rapid and accurate neuroimaging. Non-contrast computed tomography (CT) is the primary modality to rule out hemorrhagic stroke, while CT angiography or magnetic resonance imaging (MRI) with diffusion-weighted imaging (DWI) is essential for detecting ischemic lesions and identifying large-vessel occlusions [79,80]. MRI is particularly sensitive in identifying early ischemic changes, which may not be apparent on CT scans [79,80,81].

Additional diagnostic tools, such as transesophageal echocardiography (TEE) or carotid Doppler ultrasound, help identify embolic sources, particularly in cardioembolic stroke or atherosclerotic disease. Continuous cardiac monitoring is crucial in patients with suspected AF as a source of embolism [45,82].

### 3.3. Biomarkers in Stroke

Unlike ACS, stroke biomarkers are still emerging as diagnostic tools. High-sensitivity C-reactive protein (hs-CRP), fibrinogen, and D-dimer are increasingly studied as potential markers of stroke risk, reflecting underlying inflammation and coagulation abnormalities [83,84]. Biomarkers such as glial fibrillary acidic protein (GFAP) and S100 calcium-binding protein B (S100B) may help differentiate ischemic from hemorrhagic stroke, but their clinical application remains limited [85,86]. Recent studies have highlighted that inflammatory biomarkers, such as interleukin-6 (IL-6) and (hsCRP), are associated with an increased risk of recurrent cardiovascular events following ischemic stroke or transient ischemic attack. An analysis of 8420 patients demonstrated that elevated baseline levels of IL-6 and hsCRP were independently correlated with a higher risk of major adverse cardiovascular events (MACE) and recurrent stroke. Specifically, comparing the highest to the lowest quartile, IL-6 exhibited a risk ratio (RR) of 1.35 for MACE and 1.33 for recurrent stroke, while hsCRP showed an RR of 1.31 for MACE [87]. Furthermore, this meta-analysis revealed that IL-6 was associated with an increased risk of MACE in stroke subtypes related to large artery atherosclerosis, small vessel occlusion, and stroke of undetermined cause, with RR values of 2.30, 1.71, and 1.78, respectively. Conversely, hsCRP was associated with an increased risk of MACE only in strokes of undetermined cause, with an RR of 1.45 [87]. In summary, both IL-6 and CRP are inflammatory biomarkers with significant predictive value for the risk of recurrent vascular events after stroke.

### 3.4. Future Directions in Biomarkers and Diagnostics

Integrating advanced imaging with novel biomarkers holds promise for enhancing the precision of ACS and stroke diagnosis. Multi-modality approaches, such as combining cTn with imaging-based myocardial perfusion assessments or using genetic and proteomic profiling, could refine risk stratification and therapeutic strategies [88,89]. In stroke, the exploration of blood-based biomarkers for early identification of large-vessel occlusion and rapid differentiation of stroke subtypes is an area of active research [90,91]. 

## 4. Treatment and Acute Management of ACS and Stroke

Effective treatment strategies for ACS and stroke aim to promptly restore perfusion, minimize damage and prevent recurrent events. While the therapeutic approaches for ACS and stroke may differ due to their distinct pathophysiological mechanisms, both conditions rely on timely intervention and evidence-based pharmacological regimens to improve outcomes. In particular, the prevention therapy of ischemic and hemorrhagic stroke in patients with acute myocardial infarction is complex and needs a personalized approach that carefully weighs the risks and benefits of various treatments, ensuring tailored care for optimal patient outcomes.

### 4.1. Treatment of Acute Coronary Syndrome

**Figure 4:**
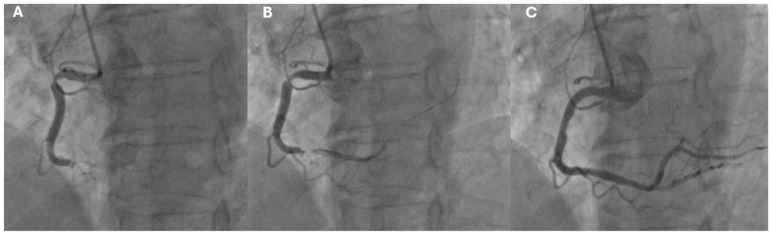
Coronary angiographic findings in a patient with inferior STEMI. (**A**) Initial angiography showing a thrombotic distal occlusion and a severe proximal stenosis of the right coronary artery. (**B**) Partial restoration of distal coronary flow following the passage of the coronary guidewire, with a visible radiolucent area at the site of the distal occlusion, suggestive of coronary thrombosis. (**C**) Final angiographic result after primary PCI with implantation of two drug-eluting stents.

**Figure 5:**
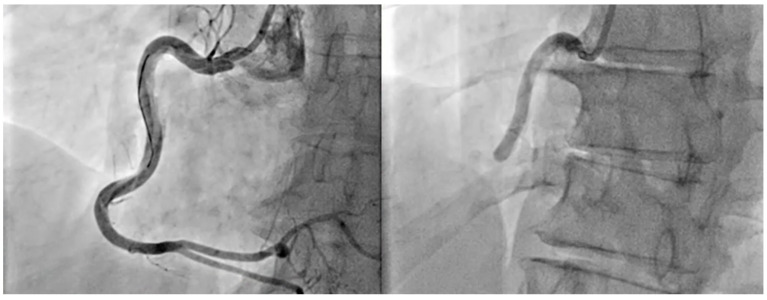
Spontaneous coronary artery dissection (SCAD) of the right coronary artery: a rare but important cause of acute coronary syndrome, predominantly affecting women. Image illustrates characteristic angiographic findings of SCAD, highlighting the arterial wall dissection and lumen narrowing, which can lead to myocardial ischemia.

The management of ACS depends on the specific type (STEMI or NSTEMI/UA) and the patient’s clinical profile. In the STEMI setting, the immediate goal is reperfusion, achieved through primary PCI within 60 or 90 min (according to the center in which the patient receives the diagnosis) from first medical contact, or, if unavailable, fibrinolytic therapy administered within 30 min [9,25] (Figure 4 and Figure 5).

For NSTEMI and UA, an invasive strategy is often pursued based on patient’s clinical features and risk stratification using scores like GRACE or TIMI. DAPT with aspirin and a P2Y12 inhibitor (clopidogrel, ticagrelor, or prasugrel) is the cornerstone of treatment, complemented by anticoagulation in the peri-procedural setting [37]. Additional measures include high-dose statins, beta-blockers, and angiotensin-converting enzyme inhibitors (ACEIs) or angiotensin receptor blockers (ARBs) for secondary prevention [9,37,92,93].

Antiplatelet therapy is pivotal in the management of ACS patients to reduce the risk of ischemic events, including stroke. However, its use is associated with an inherent trade-off: while it significantly lowers the likelihood of thrombotic events, it also increases the risk of bleeding, especially in patients at high bleeding risk due to baseline comorbidities.

More novel antiplatelet agents, such as prasugrel and ticagrelor, offer superior platelet inhibition compared to clopidogrel. Prasugrel, as demonstrated in the TRITON-TIMI 38 trial, has a more consistent and potent inhibitory effect on platelet aggregation, leading to a significant reduction in the risk of myocardial infarction, stent thrombosis, and ischemic stroke compared to clopidogrel [94]. Similarly, ticagrelor, evaluated in the PLATO trial, showed a marked reduction in death from vascular causes, MI, and stroke, outperforming clopidogrel in both STEMI and NSTEMI patients [95]. Despite these benefits, both prasugrel and ticagrelor are associated with a higher risk of major bleeding events, particularly in older patients or those with a history of hemorrhagic complications [96,97].

Clopidogrel, though less potent, remains an option for patients unable to tolerate prasugrel or ticagrelor. However, its efficacy is limited by genetic variability in the transformation of the prodrug to its active metabolite, leading to inconsistent platelet inhibition [98,99,100]. This variability not only reduces its effectiveness in preventing thrombotic events but also paradoxically increases the risk of bleeding and stent thrombosis in certain populations.

Recent trials have sought to optimize antiplatelet therapy by tailoring its intensity and duration to individual patient profiles. The TICO trial demonstrated that in patients with ACS undergoing PCI, transitioning to ticagrelor monotherapy after an initial three months of DAPT effectively reduced the risk of bleeding without increasing ischemic complications [101]. This finding supports a "de-escalation" approach to antiplatelet therapy, particularly in patients at high risk of bleeding, providing a safer and effective mean of preventing ischemic events.

The delicate balance between the ischemic benefits and bleeding risks of antiplatelet therapy is further complicated in the context of simultaneous ACS and acute IS. In these scenarios, the prioritization of treatments is critical, as the dosages and timing of therapies, such as thrombolysis and PCI, must be carefully coordinated to minimize adverse outcomes. For instance, in acute IS occurring alongside ACS, the American Heart Association/American Stroke Association (AHA/ASA) guidelines recommend administering alteplase at the stroke-specific dose and proceeding with PCI for myocardial infarction [80]. These cases highlight the necessity of individualized treatment plans that integrate the dual risks of bleeding and thrombosis.

A comprehensive evaluation of both pro-thrombotic and pro-hemorrhagic risk factors is essential to determine the most appropriate therapeutic strategy. Personalized antiplatelet regimens, informed by clinical trials and patient-specific factors, are crucial to maximizing benefit while minimizing harm in this complex patient population.

### 4.2. Treatment of Acute Ischemic Stroke 

The primary goal in managing acute IS is to restore blood flow as quickly as possible. Intravenous thrombolysis (IVT) using tissue plasminogen activator (tPA) is a cornerstone of treatment and it is most effective when administered within 4.5 h of symptom onset [101,102]. For patients with large-vessel occlusion (LVO), endovascular thrombectomy (EVT) offers another critical intervention. EVT is typically performed within 6 h of symptom onset, but advanced imaging techniques can extend the treatment window up to 24 h in selected cases, ensuring that more patients can benefit from this life-saving procedure [80,103].

Adjunctive therapies, including antiplatelet agents like aspirin (administered after 24 h post-tPA) and anticoagulation for cardioembolic stroke prevention, are critical in long-term management [80,104,105]. Blood pressure management, glucose control, and the prevention of complications such as cerebral oedema are additional priorities in the acute phase.

### 4.3. Treatment of Intracranial Hemorrhage

The management of ICH primarily aims to limit hematoma expansion and control intracranial pressure, which are critical to improving patient outcomes [106]. Blood pressure regulation plays a central role, with current guidelines recommending a target systolic pressure of 140–160 mmHg to reduce the risk of further bleeding [106]. In cases where patients are on anticoagulant therapy, reversing anticoagulation is essential. This can be achieved through the administration of prothrombin complex concentrates or specific antidotes, such as idarucizumab for dabigatran or andexanet alfa for factor Xa inhibitors [107]. For patients experiencing significant mass effect or worsening neurological symptoms, surgical options like hematoma evacuation or decompressive craniectomy may be necessary, providing relief from elevated intracranial pressure and preventing further neurological decline [106].

### 4.4. Shared Strategies and Challenges

**Figure 6:**
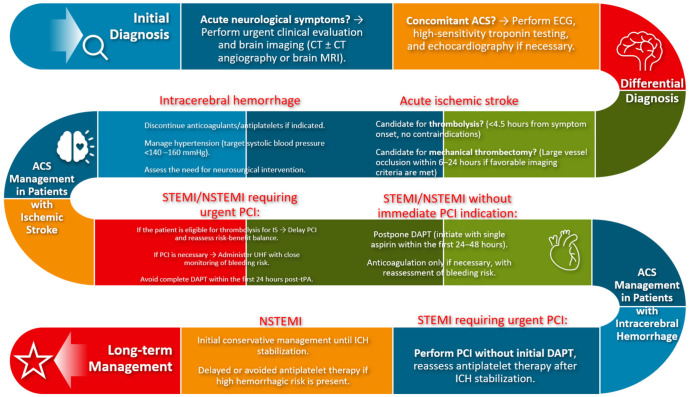
Flowchart for management of stroke in patients with ACS. ACS: Acute Coronary Syndrome, CT: Computed Tomography, ECG: Electrocardiogram, DAPT: Dual Antiplatelet Therapy, ICH: Intracerebral Hemorrhage, NSTEMI: Non-ST-Elevation Myocardial Infarction, PCI: Percutaneous Coronary Intervention, STEMI: ST-Elevation Myocardial Infarction.

Both ACS and stroke management require balancing the benefits of antithrombotic therapy against bleeding risks, particularly in patients requiring dual or triple antithrombotic therapy for coexisting conditions [108]. Novel strategies, such as tailored antithrombotic regimens guided by genetic testing or biomarkers, are being explored to optimize outcomes while minimizing adverse effects [109] (Figure 6). Additionally, integrating multidisciplinary care teams, including cardiologists, neurologists, and interventional specialists, is essential for comprehensive patient management. 

Emerging therapies, such as neuroprotective agents for stroke and new-generation antithrombotics for ACS, are under investigation to further improve survival and functional recovery [110]. Advanced imaging and AI-driven decision support systems may also enhance patient selection for interventional therapies, ensuring timely and personalized care [111].

## 5. Secondary Prevention and Risk Management in Coexisting Stroke and Myocardial Infarction

The coexistence of stroke and AMI poses unique challenges in both acute and long-term management. Balancing the risks of thrombosis and hemorrhage requires a tailored therapeutic approach to address both conditions simultaneously, as their pathophysiological mechanisms and treatment strategies may conflict.

### 5.1. Acute Management of Coexisting Stroke and Acute Myocardial Infarction

When stroke and AMI occur concurrently, the priority is to stabilize the patient and mitigate life-threatening complications. In the acute setting, treatment focuses on achieving reperfusion while carefully balancing bleeding risks, particularly in ischemic stroke complicated by thrombolytic or anticoagulant therapy.

In acute IS, IVT with tPA may be indicated if the patient meets standard eligibility criteria, provided that there are no contraindications such as active bleeding or a large MI with a high risk of hemorrhage. However, in patients with significant AMI requiring urgent PCI, fibrinolytic therapy may need to be deferred or carefully timed to avoid exacerbating stroke-related complications [112].

For STEMI, primary PCI remains the gold standard, even in the presence of acute stroke [9]. Antiplatelet therapy, typically aspirin and a P2Y12 inhibitor (e.g., clopidogrel or ticagrelor), is administered to prevent stent thrombosis, though careful consideration is needed to mitigate the risk of hemorrhagic transformation of the stroke. Anticoagulation with unfractionated heparin is often used during PCI, with close monitoring for bleeding.

In cases of hemorrhagic stroke, the use of antithrombotic agents for AMI must be avoided or minimized, focusing on blood pressure control and managing intracranial pressure [9,106]. Mechanical reperfusion strategies, such as PCI without adjunctive thrombolysis, may be preferred to reduce bleeding risk [9,106].

### 5.2. Chronic Management and Long-Term Pharmacotherapy

In the chronic phase, managing both stroke and AMI requires a delicate balance between preventing ischemic recurrences and minimizing bleeding risks. Long-term therapy typically involves antiplatelets or anticoagulants, lipid-lowering agents, and neuroprotective or cardioprotective medications tailored to the underlying etiology.

For cardioembolic stroke associated with AF, long-term anticoagulation with DOACs or warfarin is of paramount importance [45,113]. If the patient has a history of AMI and requires DAPT, combining anticoagulants with antiplatelets must be carefully considered. In such cases, current guidelines recommend reducing the duration of DAPT, even to one week, to minimize bleeding risk, while maintaining lifelong single antiplatelet therapy or transitioning to monotherapy with anticoagulants [45,113]). 

Patients with non-cardioembolic stroke and coexisting AMI are typically managed with single antiplatelet therapy (e.g., aspirin) after the acute phase [37]. For those at high risk of recurrent events, a low-dose rivaroxaban regimen combined with aspirin has shown promise in reducing cardiovascular and cerebrovascular risks while limiting bleeding complications [114].

### 5.3. Lipid and Blood Pressure Management

Aggressive control of lipid levels is a cornerstone of secondary prevention in patients with stroke and AMI. High-intensity statin therapy is recommended and Ezetimibe, Bempedoic Acid and Pro-protein Convertase Subtilisin/Kexin Type 9 (PCSK9) inhibitors can be considered in patients with persistently elevated LDL cholesterol despite optimal statin therapy [115,116,117]. Blood pressure management is equally critical, with targets individualized based on the type of stroke and underlying cardiovascular conditions [115,116]. In ischemic stroke, gradual lowering of blood pressure is favored, while in hemorrhagic stroke, more aggressive blood pressure control is necessary to prevent rebleeding [80,118].

### 5.4. Integrated Multidisciplinary Care

The complexity of managing patients with simultaneous stroke and AMI underscores the need for a multidisciplinary team approach. Cardiologists, neurologists, and critical care specialists must collaborate to optimize acute interventions and long-term care. Advanced imaging, close monitoring of biomarkers and dynamic adjustment of therapeutic strategies based on patient-specific risks are essential to improve outcomes.

## 6. Discussion and Future Directions

Managing the coexistence of ACS and stroke represents a significant clinical challenge due to the complex interplay between the cardiovascular and cerebrovascular systems. The overlapping pathophysiological mechanisms, driven by shared risk factors such as atherosclerosis, inflammation and thrombosis, underscore the need for a holistic approach in diagnosis, treatment, and secondary prevention (Table 2).

### 6.1. Current Challenges in Management

The simultaneous occurrence of ACS and stroke requires clinicians to strike a balance between reducing thrombotic risk and minimizing bleeding complications. Although its usefulness, antithrombotic therapy poses a high risk of hemorrhagic complications [89]. This is especially challenging in cases of ischemic stroke with hemorrhagic transformation or ACS patients at high bleeding risk due to intracranial pathology [17,119].

The lack of data from robust, large-scale clinical trials specifically addressing the coexistence of these conditions is an emerging problem: most guidelines for stroke and ACS come from independent studies, making it difficult to establish evidence-based protocols [9,120]. Furthermore, heterogeneity in patient presentations and comorbidities complicates the standardization of care.

### 6.2. Advances in Diagnosis and Risk Stratification

Recent advances in imaging and biomarker research offer new opportunities for early and precise diagnosis. Multi-modal imaging techniques, such as high-resolution MRI for stroke and hybrid PET/CT for coronary artery disease, can provide comprehensive assessments of both conditions [121,122]. Meanwhile, emerging biomarkers—such as high-sensitivity cardiac troponins (hs-cTn) for ACS and GFAP for stroke—could facilitate rapid differentiation of ischemic and hemorrhagic events, aiding in timely therapeutic decisions [85,86,88,123].

The use of artificial intelligence (AI) in the diagnosis of ACS and stroke is rapidly enhancing diagnostic accuracy and clinical management [124]. According to recent studies, AI algorithms outperform conventional risk scores in detecting myocardial infarction, offering greater precision and faster response times [124,125].

In the context of stroke, AI techniques, including classical machine learning and deep learning, are increasingly being applied in diagnostic imaging analysis. AI is utilized to identify early signs of ischemia, detect large vessel occlusions, assess perfusion deficits, and predict functional outcomes. Convolutional neural networks, for instance, have demonstrated the ability to detect stroke signs in MRI and CT scans with up to 97% accuracy in some studies [124,125]. Moreover, the implementation of machine learning models has improved risk stratification and reduced the time required for clinical assessment, facilitating more timely and personalized therapeutic decisions.

However, the clinical adoption of these models requires extensive validation across large multicenter cohorts to ensure the reliability and generalizability of predictions. The integration of AI into diagnostic protocols for ACS and stroke could represent a significant breakthrough in optimizing patient management, with important implications for both prognosis and healthcare system efficiency.

### 6.3. Future Directions in Treatment

Innovations in pharmacotherapy are likely to shape future management approaches for patients with coexisting ACS and stroke. Antithrombotic therapies with improved safety profiles, such as low-dose anticoagulant-antiplatelet combinations, may reduce thrombotic events without significantly increasing bleeding risks. Trials evaluating novel agents, such as Factor XI inhibitors, show promise in achieving this balance by selectively targeting coagulation pathways involved in thrombosis while sparing those critical for hemostasis [126,127].

Neuroprotective therapies are another area of active research. Agents targeting oxidative stress, inflammation, and excitotoxicity have the potential to reduce ischemic damage in the brain and heart, improving outcomes in patients with simultaneous events [110]. Ongoing trials, such as the ESCAPE-NA1 study, are evaluating such approaches in acute ischemic stroke, with implications for broader cardiovascular applications [126].

### 6.4. Integrated Care Models

The future of managing ACS and stroke lies in integrated, multidisciplinary care models. Cardiologists, neurologists, and critical care specialists must collaborate to optimize acute interventions and long-term management [128]. Comprehensive care pathways that incorporate shared decision-making, patient education and follow-up protocols are essential for improving adherence to secondary prevention strategies and reducing recurrent events [128].

Incorporating digital health technologies, such as wearable devices for continuous monitoring of AF or remote blood pressure monitoring, can enhance early detection of risk factors and ensure timely intervention [129]. These tools also facilitate patient engagement, empowering individuals to actively participate in their care.

### 6.5. Bridging Knowledge Gaps

To advance the field, future research must prioritize large-scale, randomized clinical trials focusing on patients with concurrent ACS and stroke. These studies should address key questions such as the optimal timing and combination of antithrombotic therapies, the role of advanced imaging in treatment selection, and the efficacy of novel pharmacological agents in this unique patient population. Establishing robust registries and leveraging real-world data will also provide valuable insights into outcomes and best practices.

## 7. Conclusions

The coexistence of ACS and stroke presents significant clinical challenges due to their shared risk factors and overlapping therapeutic needs. Acute management requires a careful balance between minimizing thrombotic risks and preventing hemorrhagic complications, particularly in the context of dual antiplatelet or anticoagulation therapy. Advances in diagnostics, including biomarkers and multimodal imaging, have enhanced precision in identifying high-risk patients and tailoring treatment strategies.

Long-term secondary prevention is critical, with lipid-lowering therapies, blood pressure control, and antithrombotic regimens forming the backbone of care. Emerging pharmacological agents, such as Factor XI inhibitors, and neuroprotective strategies offer hope for improving outcomes in patients with coexisting conditions.

Moving forward, an integrated approach involving cardiology, neurology, and primary care teams is essential to optimize management. Digital health innovations, including wearable technology and AI-driven decision support, are likely to play a key role in enhancing monitoring and adherence to preventive measures. However, substantial gaps remain in evidence-based guidelines for concurrent ACS and stroke, underscoring the need for large-scale trials and robust clinical registries. Addressing these challenges will pave the way for improved outcomes in this complex patient population.

## Figures and Tables

**Table 2:** Key clinical and therapeutic aspects of acute coronary syndrome (ACS) and stroke.

Aspect	Acute Coronary Syndrome (ACS)	Stroke	Shared Considerations
Primary pathophysiology	Atherosclerotic plaque rupture leading to thrombotic occlusion of coronary arteries	Ischemic stroke: Thrombosis or embolism; Hemorrhagic stroke: Rupture of intracranial vessels	Atherosclerosis, inflammation, endothelial dysfunction, thrombosis
Common risk factors	Hypertension, diabetes, smoking, dyslipidemia, atrial fibrillation (AF)	Hypertension, diabetes, smoking, AF, carotid atherosclerosis	Shared cardiovascular risk factors require integrated prevention strategies
Acute management	- Primary PCI for STEMI < 90 min - DAPT (aspirin + P2Y12 inhibitor) - Anticoagulation (UFH/LMWH) in selected cases - Beta-blockers, ACE inhibitors, statins	- IV thrombolysis (tPA) if eligible - Mechanical thrombectomy for large vessel occlusion - Antiplatelet therapy after 24 h (aspirin ± clopidogrel) - Blood pressure control	Timing of antithrombotic therapy must be balanced against bleeding risks
Antithrombotic therapy	- DAPT for ACS (aspirin + ticagrelor/prasugrel/clopidogrel) - Triple therapy (DAPT + anticoagulation) in AF with ACS	- Antiplatelets for non-cardioembolic stroke (aspirin, clopidogrel) - Anticoagulation for cardioembolic stroke (DOAC/warfarin)	Stroke prevention in AF patients with prior ACS requires tailored therapy to balance ischemic vs. bleeding risk
Procedural risks	Stroke risk from PCI/CABG due to embolization	Hemorrhagic transformation risk with reperfusion therapies (tPA, thrombectomy)	Individualized risk assessment for interventions in patients with dual pathology
Long-term prevention	- Lifestyle modification - Statins (high-intensity for ASCVD)/Ezetimibe/Bempedoic Acid/PCSK9 inhibitors - ACE inhibitors/ARBs for blood pressure control - Smoking cessation	- Antiplatelets (aspirin ± clopidogrel) - Blood pressure control (target depends on stroke type) - Lipid management with statins/Ezetimibe/Bempedoic Acid/PCSK9 inhibitors	Comprehensive cardiovascular prevention strategies are required for patients with prior ACS or stroke

## Data Availability

Not applicable.

## Abbreviations

The following abbreviations are used in this manuscript:

ACEIs	angiotensin-converting enzyme inhibitors
ACS	acute coronary syndrome
AF	atrial fibrillation
AHA	American Heart Association
AI	artificial intelligence
AMI	acute myocardial infarction
ARBs	angiotensin receptor blockers
ASA	American Stroke Association
ASCVD	atherosclerotic cardiovascular disease
CABG	coronary artery bypass grafting
cTnI	cardiac troponin
CT	computed tomography
DALYs	disability-adjusted life years
DAPT	dual antiplatelet therapy
DOACs	direct oral anticoagulants
DWI	diffusion-weighted imaging
ECG	electrocardiogram
EVT	endovascular thrombectomy
GFAP	glial fibrillary acidic protein
hs-CRP	high-sensitivity C-reactive protein
hs-cTn	high-sensitivity troponin assays
IL-6	interleukin-6
IS	ischemic stroke
IVT	intravenous thrombolysis
LMWH	low molecular weight heparin
LVEF	left ventricular ejection fraction
LVT	left ventricular thrombus
LVO	large-vessel occlusion
MACE	major adverse cardiovascular events
MI	myocardial infarction
MRI	magnetic resonance imaging
NIS	National Inpatient Sample
NSTEMI	Non ST-Segment Elevation Myocardial Infarction
PCI	percutaneous coronary intervention
PCSK9	Subtilisin/Kexin Type 9
RR	risk ratio
S100B	S100 calcium-binding protein
SCAD	Spontaneous coronary artery dissection
STEMI	ST-Segment Elevation Myocardial Infarction
tPA	tissue plasminogen activator
TTE	transesophageal echocardiography
UA	unstable angina
UI	uncertainty interval
UFH	unfractionated heparin
URL	upper reference limit
WMA	wall motion abnormalities
